# The antifungal plant defensin AtPDF2.3 from *Arabidopsis thaliana* blocks potassium channels

**DOI:** 10.1038/srep32121

**Published:** 2016-08-30

**Authors:** Kim Vriens, Steve Peigneur, Barbara De Coninck, Jan Tytgat, Bruno P. A. Cammue, Karin Thevissen

**Affiliations:** 1 Centre of Microbial and Plant Genetics, KU Leuven, Kasteelpark Arenberg 20, 3001 Heverlee, Belgium; 2 Toxicology and Pharmacology, University of Leuven, O&N 2, Herestraat 49, P.O. Box 922, 3000, Leuven, Belgium; 3 VIB Department of Plant Systems Biology, Technologiepark 927, 9052 Ghent, Belgium

## Abstract

Scorpion toxins that block potassium channels and antimicrobial plant defensins share a common structural CSαβ-motif. These toxins contain a toxin signature (K-C_4_-X-N) in their amino acid sequence, and based on *in silico* analysis of 18 plant defensin sequences, we noted the presence of a toxin signature (K-C_5_-R-G) in the amino acid sequence of the *Arabidopsis thaliana* defensin AtPDF2.3. We found that recombinant (r)AtPDF2.3 blocks K_v_1.2 and K_v_1.6 potassium channels, akin to the interaction between scorpion toxins and potassium channels. Moreover, rAtPDF2.3[G36N], a variant with a KCXN toxin signature (K-C_5_-R-N), is more potent in blocking K_v_1.2 and K_v_1.6 channels than rAtPDF2.3, whereas rAtPDF2.3[K33A], devoid of the toxin signature, is characterized by reduced K_v_ channel blocking activity. These findings highlight the importance of the KCXN scorpion toxin signature in the plant defensin sequence for blocking potassium channels. In addition, we found that rAtPDF2.3 inhibits the growth of *Saccharomyces cerevisiae* and that pathways regulating potassium transport and/or homeostasis confer tolerance of this yeast to rAtPDF2.3, indicating a role for potassium homeostasis in the fungal defence response towards rAtPDF2.3. Nevertheless, no differences in antifungal potency were observed between the rAtPDF2.3 variants, suggesting that antifungal activity and K_v_ channel inhibitory function are not linked.

Voltage-gated potassium channels (K_v_) are a diverse family of membrane-spanning proteins that selectively transfer potassium ions across the cell membrane in both excitable and non-excitable cells. These proteins play important roles in cellular signaling processes, such as regulating heart rate and insulin secretion^1^ and are involved in diverse physiological processes, including repolarization of action potential, cellular proliferation and migration and regulating cell volume^2^. K_v_ channels are considered to be ideal pharmacological targets for the development of new therapeutic drugs to treat cancer, autoimmune diseases and cardiovascular, neurological and metabolic disorders. For instance, K_v_1.3 constitutes a promising target for treatment of autoimmune diseases, such as multiple sclerosis, as this channel is overexpressed in activated effector memory T cells^2^,^3^,^4^.

Scorpion toxins, among others, are well reported to interact with K_v_ channels. In 1999, Tytgat and colleagues suggested a general nomenclature for scorpion toxins active on K_v_ channels (α-KTxs), based on the similarity between the primary structures of those toxins^5^. Nowadays, more than 200 different scorpion toxins specific for potassium channels are divided in over 30 subfamilies, based on amino acid sequence motifs and on the location of cysteine residues that are crucial for 3D-structure^5^,^6^,^7^. Recently, it was shown that a toxin signature sequence can be assigned to α-KTxs. It has been proposed that most toxins that block K_v_ channels possess a conserved functional core composed of a key basic residue (Lysine or Arginine) associated with a key hydrophobic or aromatic residue (Leucine, Tyrosine, or Phenylalanine) within a 6.6 ± 1-Å distance. Such a functional dyad can be found in a broad range of structurally unrelated peptides from various animals, such as scorpions, cone snails, snakes, and sea anemones^8^,^9^. However, it has been reported that besides this dyad, other determinants are required for a high-affinity interaction between the toxin and its target^10^. Examples of toxins lacking a dyad but still capable of blocking K_v_ channels strongly suggest that the functional dyad on its own cannot represent the minimal pharmacophore or prerequisite for K_v_1 binding^11^. These other determinants consist of eight structurally and functionally important residues conserved across the α-KTxs family, in which six cysteines are involved in three disulfide bridges and two amino acids (Lysine and Asparagine) in a four-residue long motif around the fourth cysteine (K-C_4_-X-N) (X, any amino acid) are key functional residues of α-KTxs^12^. Mutations at these two sites (Lysine27 and Asparagine30) had the largest destabilizing effects on binding of agitoxin2, an α-KTx isolated from the venom of the scorpion *Leiurus quinquestriatus hebraeus,* to the *Shaker* potassium channel in *Drosophila*^10^,^13^. This is consistent with a toxin-channel complex model derived from solid-state nuclear magnetic resonance (NMR) studies^14^. In that study, the side chains of Asparagine30 on the toxin kaliotoxin and Aspartic acid64 on the pore helix of one chain of KcsA-K_v_1.3 (structurally equivalent to Aspartic acid431 of *Drosophila melanogaster Shaker* potassium channel or Aspartic acid361 of rat K_v_1.1) are predicted to form hydrogen bonds, whereas side chains of Lysine27 directly enter into the pore region to contact the backbone carbonyls of Tyrosine78 on the channel filter (structurally equivalent to Tyrosine445 of *D. melanogaster Shaker* K^+^ channel or Tyrosine375 of rat K_v_1.1)^14^. The functional importance of these two residues was also identified in a recent crystal structure of a K_v_ channel in complex with the α-KTx charybdotoxin, though in this complex the location of the Asparagine slightly differs from the NMR-based complex model^12^,^14^,^15^.

To date, only few K_v_ blockers are used in clinical settings. For instance, 4-aminopyridine, a K_v_1 channel blocker, was marketed as a treatment for multiple sclerosis as it improved the walking speed of patients in phase III clinical trials^16^. Brivaness, which inhibits the atrial-specific channels K_v_1.5 and K_ir_3.1/3.4, was approved in Europe as a new antiarrhythmic drug, as it was effective in terminating acute-onset atrial fibrillation^17^. Although several other K_v_ blockers entered clinical trials nowadays, and hence, await results on their efficacy in specific diseases, many K^+^ channel modulators lack specificity and have significant off-target toxicities^4^. These findings highlight the importance to identify and develop novel K_v_ blocking compounds in order to treat K_v_-related diseases.

In the search for tools to further develop novel K_v_ blocking compounds, we focused on another family belonging to the superfamily of cysteine-stabilized αβ (CSαβ) peptides, namely plant defensins. Plant defensins are small, basic, cysteine-rich peptides with antimicrobial activity against a wide range of microorganisms^18^,^19^. These peptides have been studied extensively the past decades and their antifungal activity has been well documented. They are of great interest as potential novel therapeutic agents, as they are suggested to be non-toxic for mammalian cells due to their initial interaction with microbe-specific targets that are absent in mammalian cells^20^. The extensive distribution of the common CSαβ structural motif throughout diverse organisms highlights that this relatively stable and versatile scaffold has the potential to tolerate insertions, deletions and substitutions within the structure^21^. It is the noteworthy CSαβ resemblance suggesting some similarity to scorpion toxins that block potassium channels which hinted us to investigate the possible interaction between plant defensins and K_v_ channels. Note that introduction of this scorpion toxin signature in the sequence of insect defensins, which does not contain such toxin signature, results in potassium channel blocking activity^12^.

In the present study, we analyzed the potential of several plant defensins to interact with potassium channels *in silico*, based on the presence of the potassium toxin signature sequence. Based on this analysis, we found that the *Arabidopsis thaliana* defensin AtPDF2.3 sequence contains a partial toxin sequence, and hence, we heterologously expressed this peptide, along with various AtPDF2.3 variants, either bearing a KC, a CXN or a KCXN toxin signature. We tested the ability of all these recombinant (r)AtPDF2.3 variants to block potassium channels. In addition, we investigated a possible link between ion channel inhibitory function of AtPDF2.3 and its variants, and their antifungal activity.

## Results

### *In silico* analysis points to potential ion channel activity of the *Arabidopsis thaliana* plant defensin AtPDF2.3

**Figure 1:**
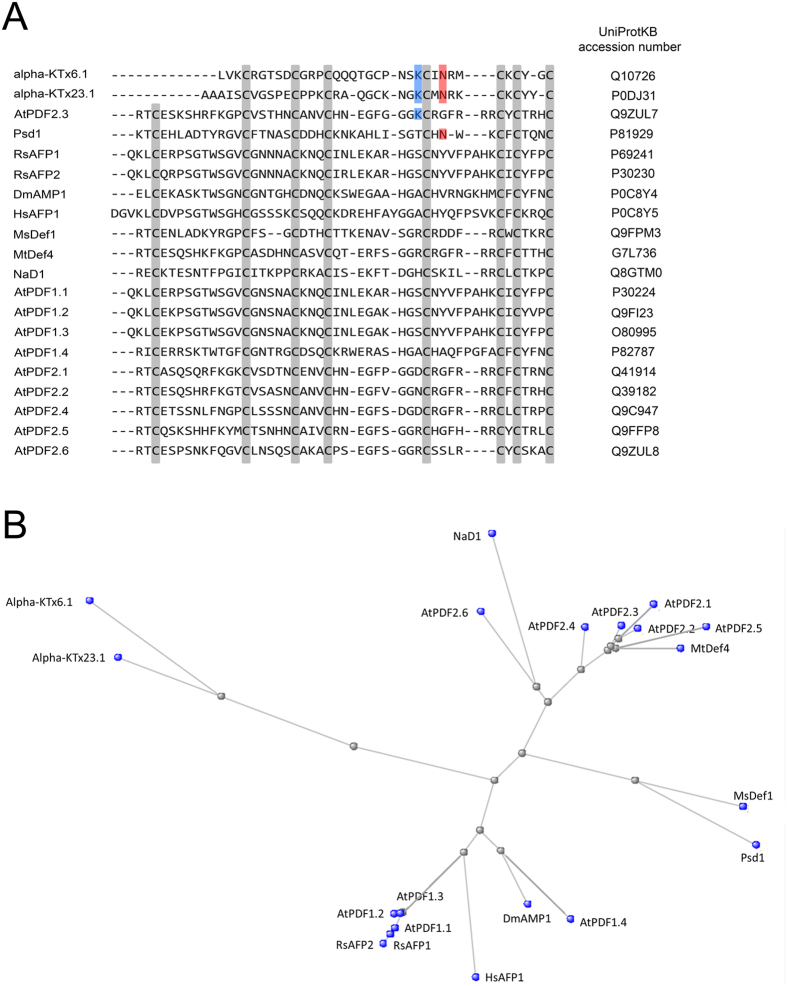
*In silico* analysis of plant defensins used in this study. (**A**) Sequence alignment between representative scorpion toxins and plant defensins. Sequences were aligned, matching the conserved cysteine residues in plant defensin sequences, using the COBALT alignment tool^24^; (−) denotes gaps in the alignment. UniProtKB accession numbers are presented. Conserved cysteine residues in plant defensin sequences are highlighted in grey; Lys27 and Asn30 present in the toxin signature are highlighted in blue and red, respectively. (**B**) Phylogenetic tree of sequences presented in (**A**) calculated by COBALT using the Fast Minimum Evolution method and Grishin distance^24^.

To investigate whether the conserved toxin signature is present in plant defensins, 18 plant defensins were aligned with representatives of the two α-KTx subfamilies that show the highest homology with these defensins, namely α-KTx6.1^22^ and α-KTx23.1^23^, using the COBALT alignment tool^24^ (Fig. 1A). The selection of plant defensins comprised defensins from *Arabidopsis thaliana* (AtPDF1.1–1.4 and AtPDF2.1–2.6^25^,^26^), *Pisum sativum* (Psd1^27^), *Raphanus sativus* (RsAFP1 and RsAFP2^28^), *Dahlia merckii* (DmAMP1^29^), *Heuchera sanguinea* (HsAFP1^29^)*, Nicotiana alata* (NaD1^30^), *Medicago sativa* (MsDef1^31^) and *Medicago truncatula* (MtDef4^32^), representing a selection of diverse plant defensins with regard to mode of action and target specificity. A phylogenetic tree comprising all the selected plant defensins as well as the two representatives of the scorpion potassium toxins is presented in Fig. 1B, pointing to the diverse set of amino acid sequences.

The α-KTx6.1, also known as Pi1, is a 35-residue toxin cross-linked by four disulfide bridges that has been isolated from the venom of the chactidae scorpion *Pandinus imperator.* Pi1 inhibits K_v_1 subtypes with lower nM (Shaker B) and even pM (K_v_1.2, K_v_1.3) affinities^33^,^34^. The α-KTx23 subfamily is represented by Vm24, a novel 36-residue K_v_1.3-specific peptide isolated from the venom of the scorpion *Vaejovis mexicanus smithi*. Vm24 inhibits K_v_1.3 channels of human lymphocytes with pM affinity^35^. Both α-KTx6.1 and α-KTx23.1 possess the toxin signature with the Lysine27 and Asparagine30 present.

As shown in Fig. 1A, the hyper-conserved and functionally crucial Lysine27 is only present in AtPDF2.3, but not in the other plant defensins analyzed in this study. In addition, Psd1 is the only defensin that contains the Asparagine30 residue. For this study, we chose to focus on AtPDF2.3 and investigated its potential interaction with potassium and sodium channels, as well as its antifungal activity. To this end, AtPDF2.3 was produced in *Pichia pastoris* and recombinant (r)AtPDF2.3 was purified using cation exchange and reversed phase chromatography. At least 70 mg/L of culture of purified rAtPDF2.3 was obtained, which was verified by LC-MS.

### rAtPDF2.3 blocks K_v_1.2 and K_v_1.6 channels

**Figure 2:**
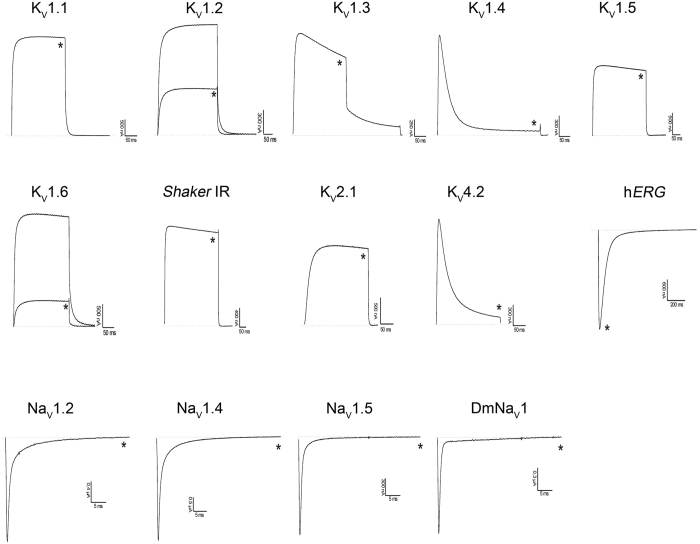
Activity of rAtPDF2.3 on ion channels expressed in *X. laevis* oocytes. Traces shown are representative of at least three independent experiments (n ≥ 3). The dotted line indicates the zero current level. The asterisk (*) distinguishes the steady-state current after application of 3 μM defensin.

rAtPDF2.3 was subjected to testing against a wide range of ion channels. The peptide’s activity was investigated on 14 cloned voltage-gated potassium channels (K_v_1.1–K_v_1.6, K_v_2.1, K_v_4.2, *Shaker* IR, and h*ERG*) and four cloned voltage-gated sodium channels (Na_v_1.2, Na_v_1.4, Na_v_1.5, and the insect channel DmNa_v_1) (Fig. 2). rAtPDF2.3 does not show activity on Na_v_ channels at 5 μM (n ≥ 4) (Supplementary Figure S1), whereas it can block K_v_1.2 and K_v_1.6 channels at 3 μM. The same concentration of rAtPDF2.3 has no effect against other K_v_ channel isoforms from the *Shaker* (K_v_1.1, K_v_1.3, K_v_1.4-K_v_1.5 and *Shaker* IR), *Shab* (K_v_2.1), *Shal* (K_v_4.2), and h*ERG* (K_v_11.1) subfamilies. These data suggest that rAtPDF2.3 acts as a toxin active on K_v_ channels, and more specifically, on K_v_1.2 and K_v_1.6 channels.

**Figure 3:**
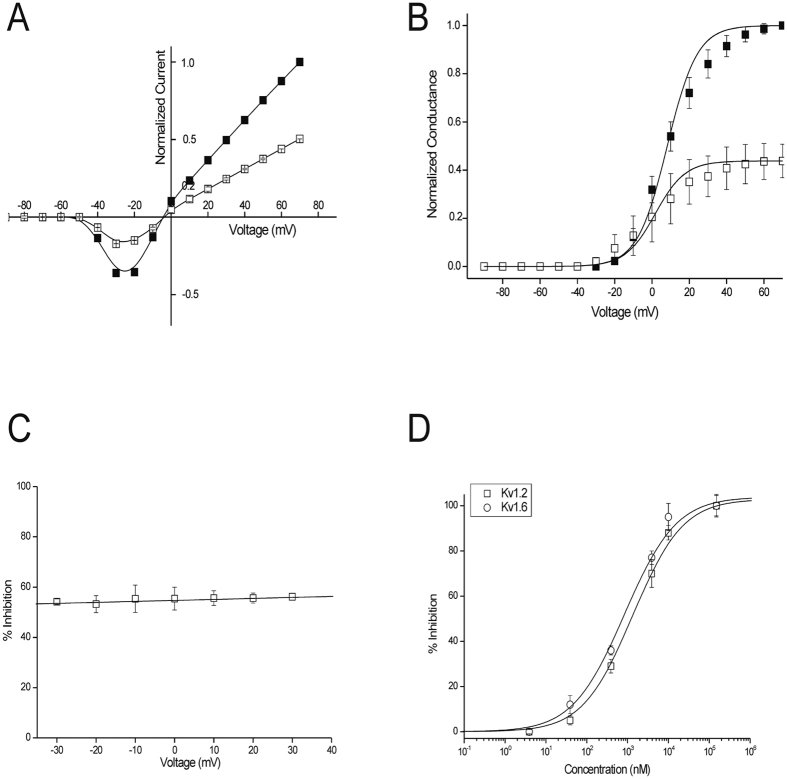
rAtPDF2.3 induces modulation of K_v_1.2 channel gating. (**A**) Current-voltage relationship in HK. Closed symbols represent control condition, open symbols after application of 2 μM rAtPDF2.3; (**B**) Conductance-voltage relationship in ND96. Closed symbols represent control conditions, open symbols after application of 2 μM rAtPDF2.3; (**C**) The percentage of inhibition at a broad range of potentials is shown. No voltage dependence of inhibition was observed; (**D**) Concentration-response curve on K_v_1.2 and K_v_1.6 channels obtained by plotting the percentage of blocked current as a function of increasing toxin concentrations.

Next, we determined dose-response curves for rAtPDF2.3’s action against the different K_v_ channels and determined IC50 values, *i.e.* the rAtPDF2.3 concentration resulting in 50% inhibition of the K_v_1.2 and K_v_1.6 channels. The corresponding IC50 values for K_v_1.2 and K_v_1.6 are 1.3 ± 0.2 μM and 978 ± 113 nM, respectively (Fig. 3D). K_v_1.2 channels were used to further investigate the characteristics of inhibition by rAtPDF2.3. The inhibition of K_v_1.2 channels induced by the defensin is not voltage-dependent as in a range of test potentials from −30 to +30 mV, no difference in the degree of block is observed (Fig. 3C). We further investigated whether the observed current inhibition is attributed to obstruction of the pore rather than to altered channel gating upon defensin binding. Application of 2 μM of rAtPDF2.3 causes 59 ± 3% and 66 ± 4% inhibition of the potassium current in ND96 (data not shown) and HK solutions, respectively (n ≥ 5) (Fig. 3D). rAtPDF2.3 does not significantly influence the reversal potential EK, as there is no significant shift in IV relationship in HK solution. EK values yield −4 ± 1 mV in control and −2 ± 1 mV after application of defensin (P > 0.05; n ≥ 4), indicating that ion selectivity is not changed (Fig. 3A). In ND96, the gV curves in control and in the presence of 2 μM defensin are characterized by V1/2 values of 8 ± 3 and 5 ± 2 mV (n ≤ 4), respectively (Fig. 3B). It can be concluded that no significant shift in the midpoint of activation occurs (*P* < 0.05) and that rAtPDF2.3 current inhibition is attributed to obstruction of the pore, rather than to altered channel gating. Furthermore, the observation that there is no difference on the percentage-induced block in ND96 or HK leads to the conclusion that channel blockage is independent of the direction of the potassium current flux and is not influenced by the extracellular concentration of potassium ions. Altogether, these experiments imply that current inhibition upon rAtPDF2.3 binding does not result from changes in the voltage dependence of channel gating. The inhibition of K_v_1.2 channels occurs rapidly and blocking is reversible because the current recovers quickly and completely upon washout (data not shown). It can thus be assumed that rAtPDF2.3 exerts its K_v_ channel inhibiting activity by physically blocking the channels, a phenomenon described previously for many K_v_ channel toxins isolated from scorpions, snakes, cone snails and sea anemones among others^36^,^37^,^38^.

### Lysine33 and Asparagine36 are crucial for K_v_ channel inhibitory activity of rAtPDF2.3

**Figure 4:**
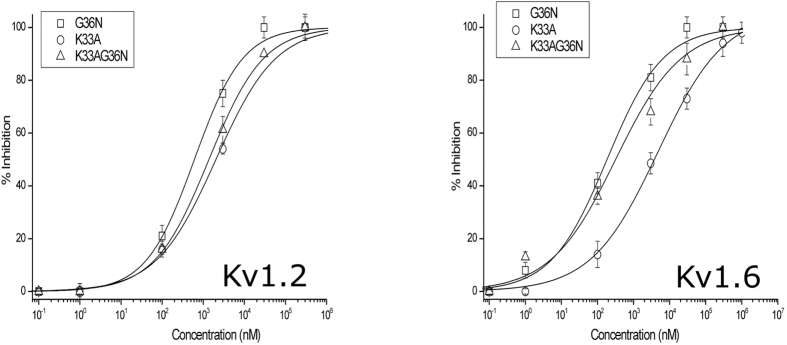
Activity of rAtPDF2.3 variants on K_v_1.2 and K_v_1.6 channels. Concentration-response curve on K_v_1.2 and K_v_1.6 channels obtained by plotting the percentage of blocked current as a function of increasing toxin concentrations. All data represent at least 3 independent experiments (n ≥ 3) and are presented as mean ± standard error. For both K_v_1.2 and K_v_1.6 channels it can be concluded that the amino acid substitutions K33A and G36N result in a decreased efficacy and in lower IC50 values, respectively.

To assess whether the presence of the two crucial amino acids, i.e. Lysine33 and Asparagine36 (based on the AtPDF2.3 sequence) previously identified in scorpion potassium toxins^12^, affects the peptide’s ability to block K_v_1.2 and K_v_1.6 channels, rAtPDF2.3 variants were produced which contain either only Asparagine36 (rAtPDF2.3[K33A][G36N]), both Lysine33 and Asparagine36 (rAtPDF2.3[G36N]) or neither of these (rAtPDF2.3[K33A]). The native rAtPDF2.3 only contains Lysine33, as indicated in Fig. 1A. The variants were subjected to electrophysiological recordings to test their ability to block K_v_1.2 and K_v_1.6 channels and dose-response curves were constructed (Fig. 4).

We found that rAtPDF2.3[G36N] has an increased potency as compared to rAtPDF2.3 in blocking K_v_ channels, with IC50 values of 611 ± 91 nM and 138 ± 38 nM for K_v_1.2 and K_v_1.6 channels, respectively, which are 2.1- and 7.1-fold decreased as compared to rAtPDF2.3, respectively. For AtPDF2.3 [K33A][G36N], the IC50 values are 1380 ± 133 nM (K_v_1.2) and 366 ± 114 nM (K_v_1.6). A reduced activity is observed for rAtPDF2.3[K33A] as compared to rAtPDF2.3, with IC50 values of 2039 ± 383 nM for K_v_1.2 and 3500 ± 235 nM for K_v_1.6 channels. In conclusion, rAtPDF2.3[G36N] is more potent in blocking K_v_1.2 and K_v_1.6 channels than rAtPDF2.3, whereas rAtPDF2.3[K33A] is characterized by reduced K_v_ channel blocking activity. These findings highlight the importance of the KCXN toxin signature in the plant defensin sequence to block potassium channels.

### rAtPDF2.3 has a broad antifungal activity spectrum

As plant defensins are reported to exert antifungal activity against a broad range of fungi and yeasts (reviewed in ref. 18,39), we further analysed the ability of rAtPDF2.3 to inhibit the growth of several plant pathogenic fungi as well as *Saccharomyces cerevisiae* (Table 1). rAtPDF2.3 shows growth inhibitory activity against all plant pathogenic fungi tested in this study, with IC50 values ranging from 1.0 to 5.8 μM. The IC50 value of rAtPDF2.3 against the yeast *S. cerevisiae* is 8.1 ± 0.9 μM.

### K_v_ channel inhibitory activity and antifungal activity seem not linked

As we showed that K_v_ channel inhibitory activity is affected by the presence of Lysine33 and Asparagine36 in the rAtPDF2.3 sequence, we further investigated whether the presence of these amino acids affects the antifungal activity as well. To this end, *F. graminearum* was challenged with the rAtPDF2.3 variants in a growth inhibitory assay, as *F. graminearum* is one of the most susceptible fungus in the panel of yeast and fungal species assessed in this study. No significant differences are found in the antifungal activity of the rAtPDF2.3 variants and that of rAtPDF2.3 against *F. graminearum*, suggesting that K_v_ channel inhibitory activity and antifungal activity are not linked.

### Potassium transport is involved in rAtPDF2.3 antifungal action against yeast

Our above data indicate that rAtPDF2.3 blocks K_v_1.2 and K_v_1.6 voltage-gated potassium channels, expressed in *X. laevis* oocytes. In yeast, potassium transport is mainly regulated by the Trk1p-Trk2p potassium transporter system^40^. In an attempt to translate the results from the electrophysiological recordings to yeast’s susceptibility to rAtPDF2.3, we analysed the rAtPDF2.3-sensitivity of ∆*trk1* and ∆*trk2* yeast strains, in addition to other knockout strains for genes that play a role in potassium homeostasis (listed in Table 2) and compared the corresponding IC50 values, i.e. the rAtPDF2.3 concentration resulting in 50% fungal growth inhibition, to that of the WT.

We found that TRK1 plays an important role in mediating tolerance towards rAtPDF2.3 in yeast, as a significantly lower IC50 value for rAtPDF2.3 is obtained for the ∆*trk1* strain as compared to the WT, i.e. 1.5 ± 0.0 μM and 8.1 ± 0.9 μM, respectively. Similarly, deletion of *HAL5*, *ARL1*, *QDR2* and *SAT4* results in increased sensitivity towards rAtPDF2.3 treatment as compared to the WT. None of the knockout strains was found more resistant to rAtPDF2.3 than WT yeast, suggesting that the tested potassium transporters are involved in generating tolerance towards rAtPDF2.3 treatment, rather than constituting its target. Note that deletion of *TRK1* or *ARL1* significantly reduces the growth rate of the corresponding knockout strains (Supplementary Figure S2), and hence, results with respect to hypersensitivity of these strains towards rAtPDF2.3 should be interpreted with care, as Trk1p and Arl1p might play an intrinsic role in yeast growth in addition to their role in potassium homeostasis. Nevertheless, these hypersensitive responses seem to be rAtPDF2.3-specific, as different responses are found when these knockout strains are challenged with rHsAFP1 (Table 3), a plant defensin that does not act on K_v_ channels (Supplementary Figure S3). None of the rAtPDF2.3-hypersensitive strains are hypersensitive to rHsAFP1, except for ∆*trk1*, which might be the result from its impaired growth. These findings indicate that involvement of potassium transport in tolerance towards rAtPDF2.3 in yeast seems to be peptide-specific and probably linked to K_v_ channel action.

## Discussion

The primary aim of this study was to broaden the scaffolds for protein engineering and drug design via the observation of a structural similarity between plant defensins and scorpion toxins. Here, we show that the native form of the plant defensin AtPDF2.3 from *Arabidopsis thaliana* can block two different subtypes of the mammalian K_v_1 voltage-gated potassium channel family. No significant changes in the voltage-dependence of steady-state activation were observed after defensin application. Furthermore, the observation that there is no difference on the percentage-induced block in ND96 or HK led to the conclusion that channel blockage is independent of the direction of the potassium current flux and is not influenced by the extracellular concentration of potassium ions. Altogether, it can thus be assumed that rAtPDF2.3 exerts its K_v_ channel inhibiting activity by physically blocking the channels, a phenomenon described previously for many K_v_ channel toxins isolated from scorpions, snakes, cone snails and sea anemones among others^36^,^37^,^38^. To date, few plant defensins were shown to interact with ion channels: the alfalfa defensin MsDef1 was shown to block the mammalian L-type Ca_v_1.2 channel in a manner similar to the antifungal toxin KP4 from *Ustilago maydis*, presumably by binding to the extracellular side of the Ca_v_1.2 pore region^31^. In addition, the pea defensin Psd1 was suggested to function as a potassium channel inhibitor, based on its electrostatic surface potential that was similar to the known potassium channel inhibitors Agitoxin 2, αKTx7.2 and OSK1 toxin^41^. In addition, the defensin-like peptide ZmES1-4 from maize was reported to interact with the intrinsic rectifying potassium channel KZM1, resulting in KZM1 channel opening and potassium influx, leading to pollen tube burst in maize^42^. Whether rAtPDF2.3 blocks plant potassium channels as in case of ZmES1-4 needs to be investigated further.

It is important to identify novel K_v_ blockers, as K_v_ channels are considered to be ideal pharmacological targets for the development of new therapeutic drugs against cancer, autoimmune diseases and cardiovascular, neurological and metabolic disorders^2^,^3^,^4^. Plant defensins can be interesting tools in this respect, as they are in general non-toxic towards human cells. Indeed, rAtPDF2.3 does not reduce cell viability in HepG2 cells up to 25 μM (Supplementary Figure S4). However, note that rAtPDF2.3’s K_v_ channel blocking activity is inferior to αKTxs in this respect and hence, direct use of rAtPDF2.3 for these purposes seems unlikely.

Recently, a specific toxin signature sequence was assigned to scorpion toxins active on potassium channels, in which the Lysine at position 27 (Lysine27) and the Asparagine at position 30 (Asparagine30) were found important for channel inhibitory activity^12^. AtPDF2.3, and by extinction other plant defensins, share a common structural fold with scorpion potassium toxins, i.e. the CSαβ motif, however, not all plant defensins possess the toxin signature sequence. Whereas the Asparagine30 is only present in Psd1, and absent in all other plant defensins analyzed in this study (Fig. 1A), the hyper-conserved and functionally crucial Lysine27 is present in AtPDF2.3 (on position 33). The sequence alignment also provides some information on the lower potency of rAtPDF2.3 compared to α-KTx. rAtPDF2.3 displays a Glycine instead of an Asparagine at the corresponding position 30 (position 36 in the AtPDF2.3 sequence). It thus can be assumed that rAtPDF2.3 forms a less stable interaction with the K_v_ channel due to the lack of stabilizing hydrogen bonds otherwise formed between Asparagine30 of the toxin and the Aspartic acid residue within the channel filter. This hypothesis is in line with the results obtained in our comparative study, in which rAtPDF2.3 variants rAtPDF2.3[G36N], rAtPDF2.3[K33A][G36N] and rAtPDF2.3[K33A] were tested for their ability to block K_v_1.2 and K_v_1.6 channels. More specifically, rAtPDF2.3[G36N], bearing the KCXN toxin signature, shows a higher activity on K_v_1.2 and K_v_1.6 channels, whereas a decreased potassium blocking activity is observed for rAtPDF2.3[K33A]. This shows that the presence of the KCXN toxin signature, containing both Lysine33 and Asparagine36, is important but not essential in potassium channel inhibitory activity. Indeed, both native rAtPDF2.3 and rAtPDF2.3[K33A][G36N], only possessing a KC or CXN toxin signature with either Lysine33 or Asparagine36, respectively, can block K_v_ channels as well. These results reinforce previous findings for scorpion toxins^12^ and broaden the knowledge on the mechanism of action of potassium channel inhibitors.

In addition, we show that there are no differences in antifungal activity between rAtPDF2.3 and its variants against *F. graminearum*, indicating that the toxin signature is not important in determining antifungal activity of this plant defensin. In order to further investigate a potential role for potassium channels in rAtPDF2.3’s antifungal activity, we investigated the effect of rAtPDF2.3 against yeast strains with deletions in genes involved in potassium transport and homeostasis. Note that orthologues of the oocyte K_v_ channels studied here have not been identified in yeast. Hence, in this study, we focused on all yeast proteins known to be involved in potassium transport and homeostasis in general. Potassium homeostasis is important in yeast, as high intracellular levels are required for many physiological processes, such as protein synthesis, enzyme activation and regulation of intracellular pH^40^,^43^. We found several potassium transport- and homeostasis-related genes to be involved in mediating tolerance towards rAtPDF2.3 treatment, but not towards rHsAFP1, a plant defensin that is not active on K_v_ channels. Hence, potassium transport in yeast seems important for governing AtPDF2.3-specific tolerance. Yet, the mechanisms underlying the observed differences between both peptides in K_v_ channel blocking activity on one hand, and antifungal tolerance mechanisms on the other hand, remain to be elucidated.

Although deletion of *TRK1* results in a reduced growth rate of this strain, and hence, Trk1p might play an intrinsic role in yeast growth in addition to potassium homeostasis, this protein is suggested to play a role in tolerance towards rAtPDF2.3, since modulators of this protein, including Hal5p, Sat4p and Arl1p, seem to gain tolerance towards rAtPDF2.3 as well. Trk1p is a component of the Trk1p-Trk2p potassium transport system in yeast, which plays a major role in potassium uptake^44^,^45^, and was previously shown to be essential for tunicamycin treatment^46^ and Histatin 5 toxicity^47^ in *C. albicans*. Similarly, Trk1p was shown to be required for fungicidal activity by lactoferrin 11, bactenecin 16 and virion-associated protein VPR 12 against *C. albicans*, pointing to Trk1p as a functional effector of these compounds^48^. In contrast, Trk1p was not required for killing of *C. albicans* by the human defensins HNP-1, hBD-2 and hBD-3^48^. The latter is in line with our observation that Trk1p is not involved in rAtPDF2.3 antifungal activity as a functional effector, but is rather part of a tolerance mechanism towards rAtPDF2.3 treatment in *S. cerevisiae*. Since *HAL5*, *SAT4* and *ARL1* are modulators of the Trk1p-Trk2p transport system, it is not surprising that the corresponding knockout strains were found hypersensitive towards rAtPDF2.3 treatment as well. More specifically, Hal5p activates the Trk1p-Trk2p transport system^49^ and hence, deletion of *HAL5* results in impairment of Trk1p-Trk2p function. Similarly, Sat4p functions as a regulator of the Trk1p-Trk2p transport system and is partially redundant with Hal5p^49^. *ARL1* encodes for a soluble GTPase that was shown to regulate potassium influx via regulation of *SAT4* and *HAL5*^50^. Deletion of *ARL1* might result in deregulation of Hal5p and Sat4p, which on its turn would lead to impairment of Trk1p-Trk2p function.

Finally, we found that also *QDR2* is involved in mediating tolerance towards rAtPDF2.3, but not rHsAFP1, treatment. Qdr2p functions as a plasma membrane transporter of many mono- and divalent cations^51^, and actively transports a variety of drugs out of the cell, such as quinidine and barban^52^. In view of the latter, it was shown that *QDR2*, in addition to *QDR3*, confers resistance to cisplatin and bleomycin in yeast^53^. In addition, *QDR2* was found to affect tolerance to oxidative stress, as strains overexpressing and lacking *QDR2* exhibited phenotypes when reactive oxygen species-producing agents, such as hydrogen peroxide and menadione, were added to the growth medium. As several plant defensins are reported to induce reactive oxygen species in yeasts (reviewed in ref. 54), and we found that *QDR2* is involved in tolerance towards rAtPDF2.3 treatment, it might well be that rAtPDF2.3 antifungal action involves the induction of oxidative stress. Whether this is the case, needs to be further investigated.

In conclusion, we show that the *A. thaliana* defensin rAtPDF2.3 interacts with K_v_1.2 and K_v_1.6 channels in a similar manner as is observed for scorpion toxins, i.e. by physically blocking the K_v_ channels. A comparative study with rAtPDF2.3 variants containing either no, a KC, a CXN or a KCXN potassium channel scorpion toxin signature revealed that Asparagine36 is important but not essential for rAtPDF2.3 K_v_ channel inhibitory activity. This is the first report of a native plant defensin interacting with mammalian K_v_ channels, and hence, this research broadens the availability of protein scaffolds, besides that of scorpion toxins, to engineer novel K_v_ blockers. It should also be taken into account that potentially also other members of the plant defensin family can affect potassium channel activity. Such information can be very relevant when medical plant defensin-based therapies are envisaged. In addition, we show that K_v_ channel inhibition and antifungal activity are not linked, as no significant differences were found in antifungal action of the rAtPDF2.3 variants. Finally, we confirm the involvement of potassium transport and/or homeostasis in rAtPDF2.3 antifungal action in yeast, more specifically by showing that several genes involved in regulating these processes confer tolerance towards rAtPDF2.3 but not to rHsAFP1, a plant defensin that does not act on K_v_ channels. It can be speculated that rAtPDF2.3 plays a dual role in plants, i.e. by (i) interacting with plant potassium channels to, for instance cause pollen tube burst, as was previously shown for ZmES1-4 from maize^42^, and by (ii) protecting the plant from fungi by its antifungal activity, which involves potassium transport and/or homeostasis. However, additional research is needed to further elucidate the mechanisms of action of rAtPDF2.3 and a potential potassium channel-dependent role for AtPDF2.3 *in planta*.

## Methods

### *In silico* analysis

Plant defensins and scorpion toxins were aligned matching their cysteine residues, using the COBALT alignment tool^24^. The sequence of all peptides analysed in this study are presented in Fig. 1A, including their corresponding accession numbers.

### Strains and reagents

*Pichia pastoris* strain X33 was used for heterologous production of AtPDF2.3. *Botrytis cinerea* (B05.10 and R16, kindly provided by Rudi Aerts, KHK Geel, Belgium)), *Verticillium dahliae* (MUCL19210), *Fusarium culmorum* (K0311; MUCL30162), *Fusarium oxysporum* (isolate 5176, kindly provided by Donald Gardiner, CSIRO, Australia) and *Fusarium graminearum* (PH-1; MUCL30161) WT and ∆*gcs*^55^ strain were used to evaluate the antifungal activity of the recombinant peptides in a fungal growth inhibitory assay^29^. *Candida albicans* (SC5314) and *Saccharomyces cerevisiae* WT (BY4741 and BY4743), ∆*ipt1*^56^, ∆*ipt1*/∆*skn1*^57^ and other yeast knockout strains tested in this study (listed in Table 2; purchased from Euroscarf; http://www.euroscarf.de) were used in a yeast growth inhibitory assay and bioscreen assays.

All culture media were purchased from LabM (UK), unless stated otherwise. For heterologous production, *P. pastoris* was cultured in YPD (1% yeast extract, 2% peptone and 2% glucose), BMGY (buffered complex glycerol medium; 1% yeast extract, 2% peptone, 1.34% yeast nitrogen base w/o amino acids (Becton Dickinson, UK), 1% glycerol, 100 mM K_3_PO_4_ pH 6, 4 × 10^−5^% biotin) or BMMY (buffered complex methanol medium; 1% yeast extract, 2% peptone, 1.34% yeast nitrogen base w/o amino acids (Becton Dickinson, UK), 0.5% methanol, 100 mM K_3_PO_4_ pH 6, 4 × 10^−5^% biotin). Plant pathogenic fungi used in the fungal growth inhibitory assay were grown in half strength PDB (1.2% potato dextrose broth). *C. albicans* and *S. cerevisiae* WT and knockout strains were grown in minimal medium (MM; 0.77 g/L complete amino acid supplement mixture (Bio 101 Systems), 6.7 g/L yeast nitrogen base w/o amino acids (Becton Dickinson, UK), 20 g/L glucose).

### Production and purification of recombinant (r) rAtPDF2.3 and rHsAFP1

Recombinant AtPDF2.3 and its variants were produced using the pPICZαA transfer vector and the *P. pastoris* expression system as previously described for recombinant HsAFP1^58^, with a minor modification: during the induction phase, 2% methanol (v/v%) was added to the culture to maintain induction of gene expression. After induction, the supernatant was collected as previously described^58^ and the presence and the molecular weight of the peptides in the supernatant was confirmed via SDS-PAGE and silver staining. The supernatant was concentrated *via* automated tangential flow filtration (Spectrum Laboratories, CA, USA) and rAtPDF2.3 was purified by cation exchange chromatography, using 75 mL SP sepharose High Performance resin (GE Healthcare, UK). For purification of the rAtPDF2.3 variants, a 5 mL SP sepharose High Performance column (GE Healthcare, UK) was used. Unbound peptides were removed via washing steps with 20 mM sodium phosphate buffers at pH 6.8. The flow rate was maintained at 5 mL/min. Elution of the peptides was carried out by a washing step with 50% (v/v%) elution buffer (20 mM sodium phosphate, 1 M sodium chloride, pH 6.8) for 10 column volumes (CV), followed by a linear gradient to 100% (v/v%) elution buffer in 15 CV, resulting in a peak at approximately 75% (v/v%) elution buffer for rAtPDF2.3, 71% for rAtPDF2.3[K33A], 80% for rAtPDF2.3[G36N] and 69% for AtPDF2.3 [K33A][G36N]. The eluted fraction was further purified by reversed phase chromatography employing a Gemini C18 250 × 10 column (Phenomenex, CA, USA) and acetonitrile (ACN) for elution of the bound peptides. The flow rate was maintained at 4.6 mL/min. Elution of the peptides was carried out by a washing step at 0% (v/v%) ACN for 1.2 CV, followed by a linear gradient to 45% (v/v%) ACN in 5.9 CV. rAtPDF2.3 was eluted at 28%, rAtPDF2.3[K33A] at 29%, rAtPDF2.3[K33A][G36N] at 26% and rAtPDF2.3[G36N] at 25%. The eluted fraction was vacuum dried by centrifugal evaporation (SpeedVac Savant, Thermo Fisher Scientific, MA, USA), re-dissolved in MilliQ water and subjected to a microbicinchoninic acid assay (Pierce, Thermo Scientific, USA) according to the manufacturer’s instructions, to determine the protein concentration. Bovine serum albumin served as a reference protein. At least 70 mg/L of culture of purified rAtPDF2.3 was obtained. The yields of the rAtPDF2.3 variants were much lower, i.e. in a range of 1.5 mg/L to 45 mg/L of culture. Recombinant HsAFP1 was produced and purified as described previously^58^.

### Expression of voltage-gated potassium channels

For the expression of the voltage-gated potassium channels (rK_v_1.1, rK_v_1.2, hK_v_1.3, rK_v_1.4, rK_v_1.5, rK_v_1.6, *Shaker* IR, hK_v_3.1, rK_v_4.3, and h*ERG*) in *Xenopus laevis* oocytes, the linearized plasmids were transcribed using the T7 or SP6 mMESSAGE-mMACHINE transcription kit (Ambion). The harvesting of stage V–VI oocytes from an anaesthetized female *X. laevis* frog was previously described^59^. Oocytes were injected with 50 nL of cRNA at a concentration of 1 ng/nL using a micro-injector (Drummond Scientific, USA). The oocytes were incubated in a solution containing (in mM): NaCl, 96; KCl, 2; CaCl_2_, 1.8; MgCl_2_, 2 and HEPES, 5 (pH 7.4), supplemented with 50 mg/L gentamycin sulfate.

### Electrophysiological recordings

Two-electrode voltage-clamp recordings were performed at room temperature (18–22 °C) using a Geneclamp 500 amplifier (Molecular Devices, USA) controlled by a pClamp data acquisition system (Axon Instruments, USA). Whole cell currents from oocytes were recorded 1–4 days after injection. Bath solution composition was ND96 (in mM): NaCl, 96; KCl, 2; CaCl_2_, 1.8; MgCl_2_, 2 and HEPES, 5 (pH 7.4) or HK (in mM): NaCl, 2; KCl, 96; CaCl_2_, 1.8; MgCl_2_, 2 and HEPES, 5 (pH 7.4). Voltage and current electrodes were filled with 3 M KCl. Resistances of both electrodes were kept between 0.7 and 1.5 MΩ. The elicited currents were filtered at 0.5 kHz and sampled at 2 kHz (for potassium currents) or filtered at 2 kHz and sampled at 20 kHz (for sodium currents) using a four-pole low-pass Bessel filter. Leak subtraction was performed using a -P/4 protocol. K_v_1.1–K_v_1.6 and *Shaker* currents were evoked by 250 ms depolarizations to 0 mV followed by a 250 ms pulse to −50 mV, from a holding potential of −90 mV. Current traces of hERG channels were elicited by applying a +40 mV prepulse for 2 s followed by a step to −120 mV for 2 s. K_v_2.1 and K_v_4.2 currents were elicited by 500 ms pulses to +20 mV from a holding potential of −90 mV. Sodium current traces were, from a holding potential of −90 mV, evoked by 100 ms depolarizations to *V*_max_ (the voltage corresponding to maximal sodium current in control conditions). In order to investigate the current–voltage relationship, current traces were evoked by 10 mV depolarization steps from a holding potential of −90 mV. To assess the concentration dependency of the toxin induced inhibitory effects, a concentration–response curve was constructed, in which the percentage of current inhibition was plotted as a function of toxin concentration. Data were fitted with the Hill equation: *y* = 100/[1 + (IC_50_/[toxin])^*h*^], were *y* is the amplitude of the toxin-induced effect, IC50 is the toxin concentration at half-maximal efficacy, [toxin] is the toxin concentration, and *h* is the Hill coefficient. GV curves were calculated from IV relationships as follows: gK = IK/(Em−EK) with EK = (RT/zF)ln[K #0]o/[K #0]i. In these equations gK represents the conductance, IK the potassium current, Em the membrane potential, EK the reversal potential, R the gas constant (8.31 J/K mol), T the temperature, z the charge of the ion (for K+ ions: z = 1), F the Faraday’s constant (96.500 C/mol), [K #0]o and [K #0]i respectively are the extracellular and intracellular K+ ion concentrations. The values of IK or gK were plotted as function of voltage and fitted using the Boltzmann equation: gK/gmax = [1 + exp(Vg−V)/k]−1, were gmax represents maximal gK, Vg is the voltage corresponding to half-maximal conductance, and k is the slope factor. Comparison of two sample means was made using a paired Student’s *t* test (*P* < 0.05). All data represent at least three independent experiments (*n* ≥ 3) and are presented as mean ± standard error.

### Antifungal activity assays

The antifungal activity of rAtPDF2.3 and its variants against a range of plant pathogenic fungi was analysed following the protocol previously described by Osborn and colleagues^29^. Briefly, a two-fold dilution series of the peptides in sterile water was prepared in 96-well plates, after which 10 μL of peptide was mixed with 90 μL of half strength PDB containing 10^4^ spores/mL of the fungus. The IC50 value, which is the concentration required for 50% growth inhibition as compared to control treatment, was determined by microscopy after 48 hours of incubation. The antifungal activity of rAtPDF2.3 and rHsAFP1 against *S. cerevisiae* and *S. cerevisiae* knockout strains was analysed according to the standard CLSI protocol M27-A3^60^ with minor modifications: an inoculum of approximately 10^6^ cells/mL was suspended in MM for rAtPDF2.3 or MMH (MM supplemented with HEPES pH 7) for rHsAFP1 and added to a two-fold dilution series of the peptides in water. The IC50 value was determined by spectrophotometry (OD_490nm_) after 24 hours of incubation. Sigmoidal curves were generated with GraphPad Prism (GraphPad Software, Inc., CA, USA), using nonlinear regression. IC50 values were derived from the whole dose-response curves. All data represent at least three independent experiments (n ≥ 3); IC50 values are presented as mean ± standard error. ANOVA followed by Dunnett post hoc test was performed to analyse statistically significant differences between the rAtPDF2.3 or rHsAFP1 IC50 values of the wild types and those of the knockout strains.

### Growth curve determination

Bioscreen assays (Bioscreen C Analyzer, Oy Growth Curves Ab Ltd, Raisio, Finland) were carried out to determine the growth rate of *S. cerevisiae* and the *S. cerevisiae* knockout strains that were found hypersensitive towards rAtPDF2.3 (Table 2). Overnight yeast cultures in YPD were washed and diluted in MM to an OD_600nm_ of 0.072 and transferred to the wells of Honeycomb plates (Oy Growth Curves Ab Ltd, Raisio, Finland). Cultures were grown for 50 hours at 30 °C, shaking, and the OD_600nm_ was measured every 15 min. Growth rates were determined employing the equation 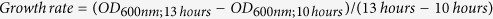, where growth was exponential, and hence in the linear range, for all strains tested. Data represent biological triplicates (n = 3) with three technical replicates each, and are presented as mean ± standard error. ANOVA followed by Dunnett post hoc test was performed to analyse statistically significant differences between the growth rates of the BY4741 WT and the knockout strains, respectively.

### rAtPDF2.3 toxicity in HepG2 cells

HepG2 cells were seeded at 10.000 cells/well in 96-well plates and incubated for 24 hours. Subsequently, cells were treated either with water (untreated) or rAtPDF2.3 (0.01 μM–23.5 μM) for 24 hours after which cell viability was determined using the ‘Cell Proliferation Kit II (XTT) as described previously^61^. ANOVA followed by Dunnett post hoc test was performed to analyze statistically significant differences between untreated and rAtPDF2.3-treated samples.

## Additional Information

**How to cite this article**: Vriens, K. *et al.* The antifungal plant defensin AtPDF2.3 from *Arabidopsis thaliana* blocks potassium channels. *Sci. Rep.* **6**, 32121; doi: 10.1038/srep32121 (2016).

## Supplementary Material

Supplementary Information

## Figures and Tables

**Table 1:** IC50 values for rAtPDF2.3 against yeast and plant pathogenic fungi.

Microorganism	IC50 (μM)
*Botrytis cinerea* B05.10	5.8 ± 0.0
*Botrytis cinerea* R16	5.8 ± 0.0
*Fusarium oxysporum*	4.4 ± 1.6
*Fusarium culmorum*	1.0 ± 0.4
*Verticillium dahliae*	4.4 ± 1.6
*Fusarium graminearum PH-1*	1.4 ± 0.0
*Saccharomyces cerevisiae BY4741*	8.1 ± 0.9

Fungi and yeast were treated with a concentration range of rAtPDF2.3 for 48 or 24 hours, respectively, after which the IC50 values were determined microscopically for fungi and spectrophotometrically for yeast. IC50, the concentration required for 50% growth inhibition as compared to control treatment after either 24 hours for yeast, or after 48 hours for fungi. Data of at least three independent experiments are shown (n ≥ 3).

**Table 2:** IC50 values for rAtPDF2.3 against *S. cerevisiae* wild type (WT) and knockout mutants.

Microorganism	IC50 (μM)	*P*-value
*Saccharomyces cerevisiae* BY4741 *WT*	8.1 ± 0.9	NA
*Saccharomyces cerevisiae* BY4743 *WT*	3.3 ± 0.0	NA
*Saccharomyces cerevisiae* BY4741 *∆trk1*	1.5 ± 0.0	<0.0001
*Saccharomyces cerevisiae* BY4741 *∆trk2*	7.0 ± 0.6	0.5862
*Saccharomyces cerevisiae* BY4741 *∆hal5*	4.0 ± 0.4	<0.0001
*Saccharomyces cerevisiae* BY4741 *∆prm6*	7.6 ± 0.2	0.9958
*Saccharomyces cerevisiae* BY4741 *∆arl1*	5.7 ± 0.2	0.0033
*Saccharomyces cerevisiae* BY4741 *∆qdr2*	2.9 ± 0.2	<0.0001
*Saccharomyces cerevisiae* BY4741 *∆sat4*	5.1 ± 0.1	0.0003
*Saccharomyces cerevisiae* BY4741 *∆vhc1*	8.0 ± 0.2	>0.9999
*Saccharomyces cerevisiae* BY4741 *∆ppz2*	8.6 ± 0.5	0.9868
*Saccharomyces cerevisiae* BY4741 *∆tok1*	7.7 ± 0.5	0.9995
*Saccharomyces cerevisiae* BY4741 *∆nha1*	9.1 ± 0.7	0.2161
*Saccharomyces cerevisiae* BY4741 *∆ppz1*	10.1 ± 0.5	0.0120
*Saccharomyces cerevisiae* BY4741 *∆kch1*	8.9 ± 0.3	0.8144
*Saccharomyces cerevisiae* BY4741 *∆kkq8*	7.1 ± 0.1	0.7009
*Saccharomyces cerevisiae* BY4743 *∆frq1*	3.1 ± 0.1	0.0935

WT and knockout yeast strains were treated with a concentration range of rAtPDF2.3 for 24 hours, after which the IC50 values were determined spectrophotometrically; IC50, the concentration required for 50% growth inhibition as compared to control treatment. Mean ± SEM is shown of at least three independent experiments (n ≥ 3). ANOVA followed by Dunnett post hoc test was performed to analyse significant differences between the effect of rAtPDF2.3 on BY4741 WT yeast and knockout mutants; the adjusted *P*-values are presented. Unpaired Student’s t-test was performed to analyse significant differences between the effect of rAtPDF2.3 on BY4743 WT and the *FRQ1* knockout mutant. NA, not applicable.

**Table 3:** IC50 values for rHsAFP1 against *S. cerevisiae* wild type (WT) and knockout mutants.

Microorganism	IC50 (μM)	*P*-value
*Saccharomyces cerevisiae* BY4741 *WT*	4.8 ± 0.1	NA
*Saccharomyces cerevisiae* BY4741 *∆trk1*	2.8 ± 0.1	<0.0001
*Saccharomyces cerevisiae* BY4741 *∆hal5*	4.6 ± 0.0	0.1073
*Saccharomyces cerevisiae* BY4741 *∆arl1*	5.2 ± 0.1	0.0281
*Saccharomyces cerevisiae* BY4741 *∆qdr2*	4.8 ± 0.0	0.4534
*Saccharomyces cerevisiae* BY4741 *∆sat4*	4.7 ± 0.1	0.1865

WT and knockout yeast strains were treated with a concentration range of rHsAFP1 for 24 hours, after which the IC50 values were determined spectrophotometrically; IC50, the concentration required for 50% growth inhibition as compared to control treatment. Mean ± SEM is shown of at least three independent experiments (n ≥ 3). ANOVA followed by Dunnett post hoc test was performed to analyse significant differences between the effect of rHsAFP1 on WT yeast and knockout mutants; the adjusted *P*-values are presented. NA, not applicable.
