# Supplementary material for: The antifungal plant defensin AtPDF2.3 from Arabidopsis thaliana blocks potassium channels
Source: Sci Rep. 2016 Aug 30;6:32121. doi: 10.1038/srep32121 (PMC5004176; doi:10.1038/srep32121)
Supplement: Supplementary Information [file srep32121-s1.pdf]

Supplementary information

The antifungal plant defensin AtPDF2.3 from *Arabidopsis thaliana* blocks potassium channels

Kim Vriens[1], Steve Peigneur[2], Barbara De Coninck[1,3], Jan Tytgat[2], Bruno P.A. Cammue[1,3,*], Karin Thevissen[1]

[1]Centre of Microbial and Plant Genetics, KU Leuven, Kasteelpark Arenberg 20, 3001 Heverlee, Belgium

[2]Toxicology and Pharmacology, University of Leuven, O&N 2, Herestraat 49, P.O. Box 922, 3000, Leuven, Belgium

[3]VIB Department of Plant Systems Biology, Technologiepark 927, 9052 Ghent, Belgium

*bruno.cammue@biw.vib-kuleuven.be

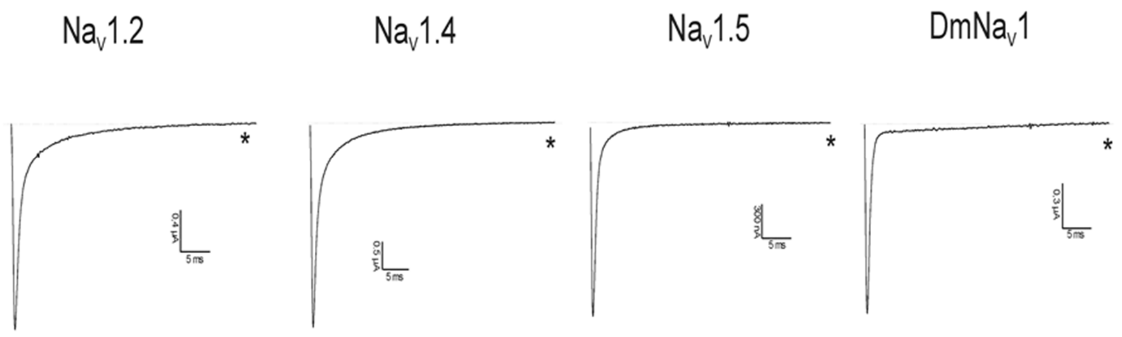

Supplementary Figure S1 Activity of rAtPDF2.3 on ion channels expressed in *X. laevis* oocytes. Traces shown are representative of at least three independent experiments (n ≥ 3). The dotted line indicates the zero current level. The asterisk (*) distinguishes the steady-state current after application of 5 µM defensin.

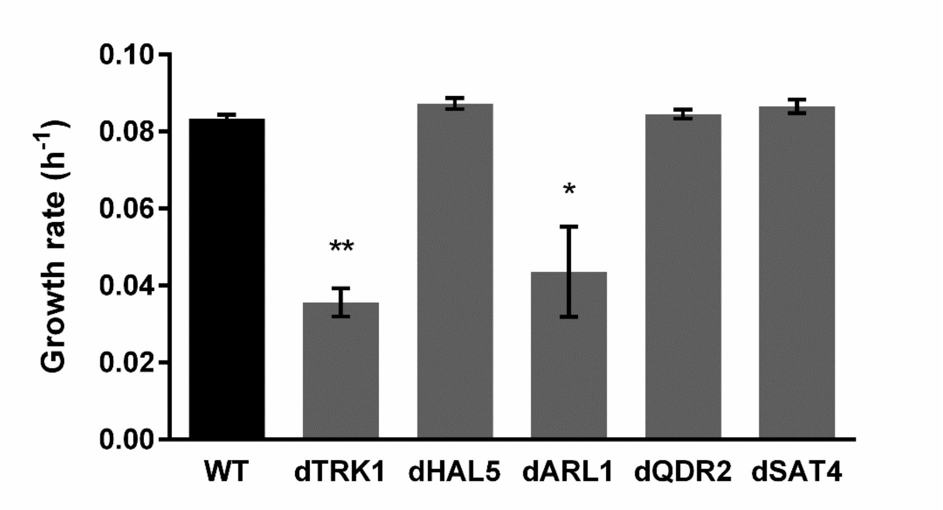

Supplementary Figure S2 Growth rates of *S. cerevisiae* BY4741 WT and knockout strains used in this study. Bioscreen assays were carried out to determine the growth rate of *S. cerevisiae* and the *S*. *cerevisiae* knockout strains that were found hypersensitive towards rAtPDF2.3 (Table 2). Growth rates were determined employing the equation$Growth\,rate$ = $(OD_{600nm;13\ hours} - OD_{600nm;10\ hours})/(13\ hours - 10\ hours)$, where growth was exponential, and hence in the linear range, for all strains tested. Data represent biological triplicates (n = 3) with three technical replicates each, and are presented as mean ± standard error. ANOVA followed by Dunnett post hoc test was performed to analyse statistically significant differences between the growth rates of the BY4741 WT and the knockout strains, respectively. * and ** represent *P* = 0.0015 and *P* = 0.0003, respectively.

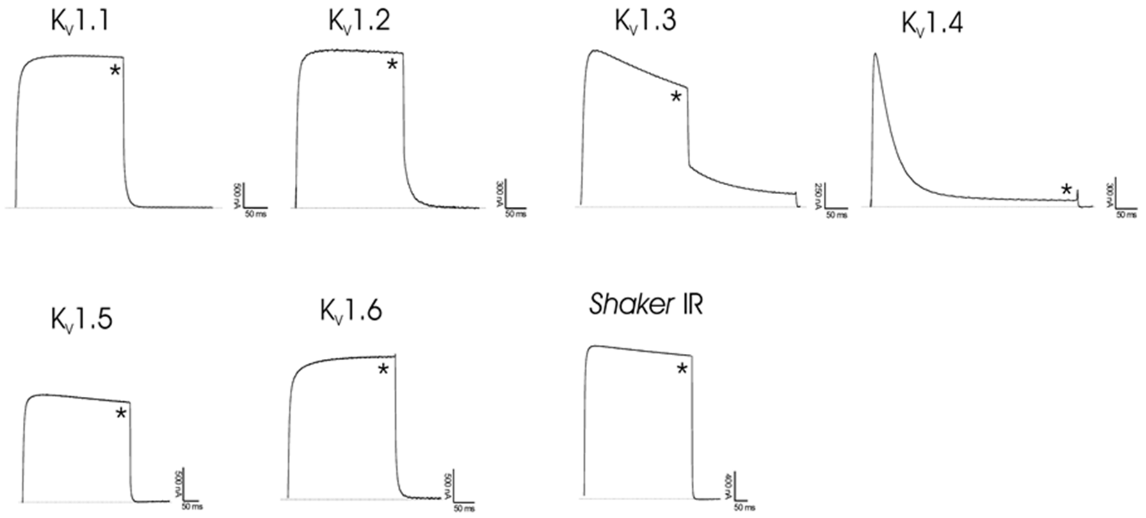

Supplementary Figure S3 Activity of rHsAFP1 on ion channels expressed in *X. laevis* oocytes. Traces shown are representative of at least three independent experiments (n ≥ 3). The dotted line indicates the zero current level. The asterisk (*) distinguishes the steady-state current after application of 3 µM defensin. Similar results were obtained for application of the defensin up to 10 µM.

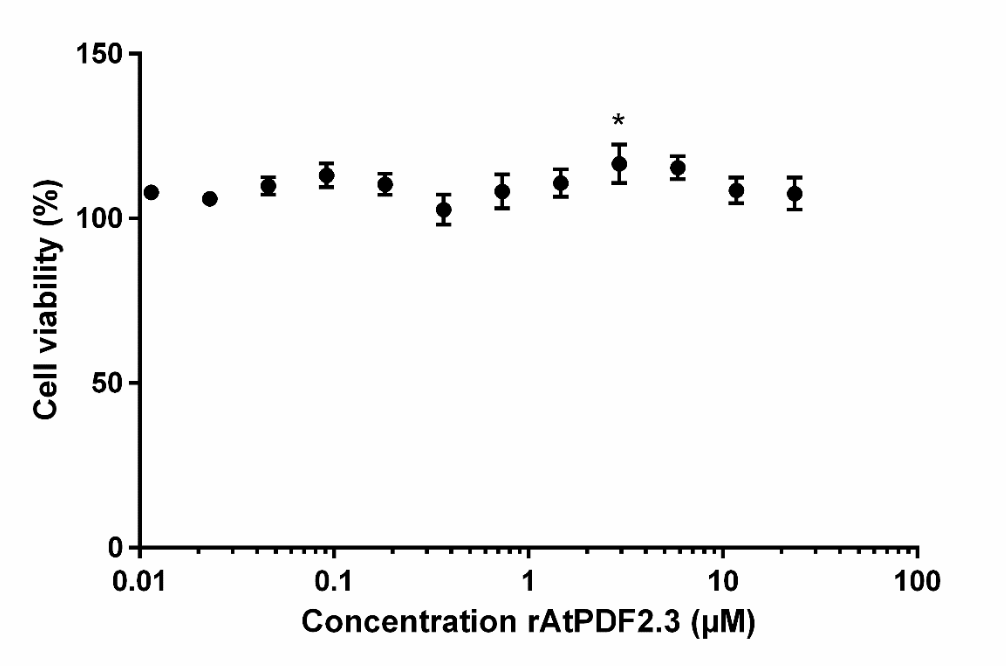

Supplementary Figure S4 Activity of rAtPDF2.3 on HepG2 cell viability. HepG2 cells were treated either with water (untreated) or rAtPDF2.3 (0.01 µM – 24.3 µM) for 24 hours, after which cell viability was determined by XTT staining. Results are expressed relative to the untreated samples. Mean ± standard error of at least two experiments in quadruplicate is shown. ANOVA followed by Dunnet post hoc test performed to analyze statistically significant differences between untreated and rAtPDF2.3-treated samples; * represents $P = 0.03$.
